# Fungal Endophytes of Tahiti Lime (*Citrus citrus* × *latifolia*) and Their Potential for Control of *Colletotrichum acutatum* J. H. Simmonds Causing Anthracnose

**DOI:** 10.3389/fbioe.2021.650351

**Published:** 2021-04-01

**Authors:** Jaider Muñoz-Guerrero, Beatriz E. Guerra-Sierra, Javier C. Alvarez

**Affiliations:** ^1^Research Group in Agro-Environmental Biotechnology and Health, MICROBIOTA, Faculty of Exact Natural and Agricultural Sciences, University of Santander, Bucaramanga, Colombia; ^2^Department of Biological Sciences, Eafit University, Medellín, Colombia

**Keywords:** mycobiota, biocontrol, citrus, phytopathogens, antagonism

## Abstract

*Colletotrichum acutatum* is one of the causal agents of anthracnose in several crops, and of post-flowering fruit drop (PFD) in citrus and key lime anthracnose (KLA). The pathogen normally attacks flowers, causing lesions only in open flowers. Under very favorable conditions, however, it can also affect flower buds and small fruits, causing complete rotting of the fruit and a premature fall, resulting in major economic crop losses. We isolated endophytic fungi from Tahiti lime to evaluate its diversity, verify its antagonistic capacity against the phytopathogen *Colletotrichum acutatum* C-100 in dual tests, and evaluate the ability of various endophytic agents to control flowers with induced anthracnose. 138 fungal isolates were obtained from 486 fragments of branches, leaves, and fruit; from which 15 species were identified morphologically. A higher isolation frequency was found in branches and leaves, with a normal level of diversity compared to other citrus species. Of the 15 morphospecies, 5 were trialed against *C. acutatum* in antagonism tests, resulting in a finding of positive inhibition. 2 endophytic fungi from the antagonism tests demonstrated high inhibition of the phytopathogen, and were thus used in *in vivo* tests with Tahiti lime flowers, applied in a spore solution. Spore solutions of two molecularly identified species, *Xylaria adscendens*, and *Trichoderma atroviride*, reduced the lesions caused by the phytopathogen in these *in vivo* tests. The finding that these endophytes react antagonistically against *C. acutatum* may make them good candidates for further biological control research in an agroindustry that requires environmental sustainability.

## Introduction

The appearance of diseases in citrus crops reduces the quantity that can be produced and sold, even more so when the products are to be exported (Tennant et al., 2009; Donkersley et al., 2018). Many citrus diseases are caused by phytopathogens, which colonize various plant tissues and affect organs such as flowers and fruits both before and after harvesting (Bazioli et al., 2019). Citrus varieties found in tropical and subtropical zones around the world are frequently infected by phytopathogenic fungi species which cause diseases such as anthracnose (Silva-Junior et al., 2014; Rhaiem and Taylor, 2016), a condition linked to *Colletotrichum* (Cannon et al., 2012; Ben Hadj Daoud et al., 2019; Mogollón et al., 2021).

Anthracnose is a serious disease in citrus, manifesting itself in symptoms such as necrosis of leaves and flowers, and the premature drop of fruit after flowering; the latter being especially harmful to the crop (Silva-Junior et al., 2014; Frare et al., 2016; Wang et al., 2021). *Colletotrichum gloeosporioides* and *Colletotrichum acutatum* are two of the species most responsible for anthracnose in citrus (Barquero et al., 2013; Ramos et al., 2016; Ben Hadj Daoud et al., 2019), including the acid lime and Tahiti lime (*Citrus citrus* × *latifolia*). Of the two varieties, Tahiti limes are more commercially common, as they are generally larger and contain more juice than key limes (Evans et al., 2014). Anthracnose results in negative phytosanitary effects and crop reductions for this important cultivar (Lima et al., 2011; Mogollón et al., 2021; Wang et al., 2021).

One possible control methodology for diseases such as anthracnose is the use of conventional fungicides (Palou et al., 2008; Bazioli et al., 2019). However, although these pesticides do help control various species of phytopathogens, their indiscriminate use also results in undesirable impacts upon the environment (Yoon et al., 2013; Price et al., 2015). Furthermore, intensive use of these agrochemicals, including in citrus crops, can lead to the generation of resistance mechanisms among phytopathogenic fungal populations (Price et al., 2015; Forcelini and Peres, 2018). Thus, biological control strategies are increasingly attractive for this type of agriculture (Pekas, 2011; Wang et al., 2018, 2020; Rojas et al., 2020), more so in the current era of sustainable agriculture, which emphasizes the reduction or elimination of fertilizers and other agrochemicals (Arora, 2018). Biological control presents a viable and environmentally friendly solution (Droby et al., 2009; De Silva et al., 2019).

Among biological control options, endophytic fungi have become a resource of interest to the agricultural world to control disease (Latz et al., 2018; De Silva et al., 2019; Ortega et al., 2020) and improve crop yields (Abo, 2019; Segaran and Sathiavelu, 2019; Rojas et al., 2020). The symbiotic interactions of these fungi, and their ability to coexist safely with the tissues of the host plant make possible the production of bioactive compounds for the control of pests and pathogens that attack plants (Selim, 2012; Kusari et al., 2013; Caruso et al., 2020). In crops, there is a pressing need to find new solutions, making the exploration of endophytic mycobiota in species such as Tahiti lime important. In the present paper, we to identify endophytic fungi in Tahiti lime (*Citrus citrus* × *latifolia* in order to evaluate their diversity and antagonistic capacity both *in vitro* and *in vivo* against a strain of phytopathogenic fungus *Colletotrichum acutatum* C-100, previously isolated from Tahiti lime flowers with anthracnose.

## Materials and Methods

### Sample Collection

Healthy Tahiti lime (*Citrus citrus* × *latifolia*) plant tissues (without visual disease) such as leaves, stems, and fruits were obtained from local farms, located in the municipality of Girón, Santander, Colombia at coordinates: 7° 1′ 31.59″ N; 73° 9′ 18.77″ W and 7° 1′ 31.78″ N; 73° 9′ 32.62″ W. The samples were transported in a cold chain and processed in the agro-environmental biotechnology laboratory (LIIBAAM_UDES) to isolate endophytic fungi within 6 h of collection. This same sampling procedure was carried out in six trips to the field.

### Endophyte Isolates

Given that each sample consisted of a different mix of leaves, fruit, and fragments of branches, the samples were initially washed in distilled water for 10 min, after which small segments of approximately 0.7 cm were cut. Thereafter, the samples were placed in separate Petri dishes for surface disinfection in a solution of alcohol (70%) for 2 min, sodium hypochlorite (2.5%) for 2 min, and then two separate washes with distilled water for 2 min each, using a protocol adapted from Kjer et al. (2010). Once the tissue fragments were disinfected, they were seeded in Petri dishes with Potato Dextrose Agar (PDA) supplemented with chloramphenicol and incubated for 10 days at 26°C. Daily inspection was carried out to select the endophytic fungi that appeared. An equal number of non-disinfected vegetal segments were prepared as a control for the disinfection process, and seeded in the mycological medium to allow for fungal growth. Fractions of fungal mycelium emerging from the plant tissues were extracted aseptically in a laminar flow cabinet to avoid contact with adjacent samples, and these fungal segments were seeded into individual Petri dishes with PDA medium. This process was carried out in triplicate to obtain pure colonies for subsequent characterization and identification.

### Colonization Frequency and Relative Isolation Frequency

The isolation frequency rate (IR) was defined as the number of endophytic fungi isolated divided by the total number of incubated fragments and expressed as a percentage. The relative frequency of isolation (FR) was calculated as the number of isolates of a given morphospecies divided by the total number of isolates, expressed as a percentage. This information was analyzed by a one-way ANOVA with a Tukey test to identify any differences between tissue types (leaves, branches and fruits).

### Characterization of Fungal Endophytes and Analysis of Diversity

Endophytic fungi were initially identified by morphospecies, based on the morphological characteristics of the colonies grown in PDA medium (colony appearance, diffusible pigments, front and back color, borders, texture, and growth rate) (Tibpromma et al., 2018; Estrada and Ramírez, 2019; da Costa Silveira et al., 2020). To determine these microscopic characteristics, preparations of the isolated endophytes were made in new PDA media, using microcultures and encouraging their sporulation. The specimens were then placed on slides with lactic acid and ethanol solution, allowing for the visualization of the mycelia using an optical microscope (Nikon eclipse-NI-U). The morphological characteristics of the isolates (hyphae and spores) were compared with previous descriptions drawn from the literature, bibliographic resources, and taxonomic keys (Barnett and Hunter, 1998; Estrada and Ramírez, 2019; da Costa Silveira et al., 2020). Isolates that did not sporulate were placed in darkness at room temperature for up to 20 days to stimulate sporulation. Those isolates that still did not produce spores were treated as sterile mycelia.

### Diversity Analysis

The diversity of endophytic morphotypes was measured using diversity indices whose parameters are the richness and relative abundance of species, and which evaluate the contribution of individuals to the community (Chao et al., 2005). Specifically, the study used the Shannon-Wiener (H′) and Simpson (D) indices. The former measures the information content per individual in samples obtained at random from a “large” community in which the total number of species (S) is known and which are sampled at random. This measurement can take values between 0 and 5 and is strongly influenced by the most abundant species (Jin et al., 2013). It is based on the following formula:

$$H^{\prime }=-\Sigma \left(Pi\times lnPi\right).$$

The latter, Simpson’s diversity index, measures the probability of finding two individuals of the same species in two successive random draws without replacement, by the following formula: (1 − D) (formula: 1 − [D = Σ(ni/n)2]), where ni, is the number of distinct species (i) and (n) is the abundance of each species in the community. The species evenness (E), which assesses the contribution of the individuals to the community, was also calculated using the following formula: E = H/lnS, where S = the number of species in the sample. The above calculations were all made using PAST 4 software and Estimates (Colwell, 2004).

### Tests for Antagonism: Endophytic Fungi vs. *Colletotrichum acutatum*

The fungal morphospecies obtained from the isolates were seeded in a medium of PDA / water agar (at a 1:3 ratio), for stressful growth. The fungi which grew in the first 5 days and demonstrated the highest growth radii were selected for the antagonism tests with the reference phytopathogenic fungi *Colletotrichum acutatum* C-100 that was isolated earlier from Tahiti lime flowers with anthracnose (Martinez et al., 2009). Mycelium discs of 5 mm diameter with both the endophytes and the phytopathogenic fungus were planted equidistantly in Petri dishes with PDA + chloramphenicol medium spaced 10 mm from the edge of the dish, and were incubated at 26°C. All of the endophyte-phytopathogen antagonism tests were performed in triplicate. They were observed daily, and the antagonistic capability of each fungus was determined, expressed as a percentage of the radial inhibition of the pathogen’s growth (PIRG-P) and the endophyte’s (PIRG-E), using the following formula for the calculation (López et al., 2017):

PIRG-Por-E(%)=(R1-R2)/R1]×100

Where R1 indicates the radial growth of the phytopathogen or the endophyte colony in control plates and R2 indicates the radial growth of the phytopathogen or the endophyte colony (in the direction of the other fungus) during the antagonism trials. To determine the type of interaction between the endophyte and the phytopathogen, the following scale was used with 5 types of interactions, adapted from López et al. (2017): (1): The two opposing fungi demonstrate similar growth and overlap; (2): the endophytic fungus outgrows the pathogenic one; (3): the phytopathogenic fungus outgrows the endophytic one; (4 and 5): mutual inhibition of the two colonies at a short distance (<2 mm) or larger distance (>2 mm), respectively. An ANOVA analysis with a Bonferroni test was applied *a posteriori* to compare the inhibition of the growth between the opposing fungi and verify which endophytic fungi had the greatest inhibitory impact on the phytopathogen.

### *In vivo* Tests on Flowers and Severity Estimation

Of the *in vitro* antagonism tests, the two morphospecies of endophytic fungi (EFTL-10 and EFTL-13) with the best inhibition results against *C. acutatum* C-100 were selected for severity tests on the flowers of Tahiti lime. Spore solutions were prepared from these two fungi with which the petals of the Tahiti lime flowers were inoculated. Mycelium sections of the two fungi were grown in flasks of liquid medium. The flasks were incubated in laboratory conditions using a rotating agitator at 140 rpm and a temperature of 25°C for 1 week to allow for the fungi to sporulate and disperse spores through the medium. From this stock solution, 5 serial dilutions were prepared to determine which was best for the inoculation. The spore density was measured in a Neubauer chamber, and the suspension was standardized using a final concentration of 1 × 10^3^ spores/mL.

The treatments were carried out in a greenhouse at a temperature of 24 ± 2°C and relative humidity of 80%, and using a completely randomized experimental design in triplicate. The Tahiti lime flowers used were in fresh anthesis (flower opening). Each set of flowers was washed with distilled water and 2.0% sodium hypochlorite for 1 min. 5 flowers were treated in the following manner:

T1: flowers inoculated with a solution of phytopathogenic spores of *Colletotrichum acutatum*;

T2: flowers inoculated with a solution of phytopathogenic spores + endophytic spore solution 1 at 12 h; T3: flowers inoculated with a solution of phytopathogenic spores + endophytic spore solution 2 at 12 h; T4: 5 flowers inoculated with distilled water as a control. All of the treatments were inspected at 12, 48, and 72 h for the final measurement of petal lesions, according to a protocol adapted from Goulin et al. (2019).

To calculate the lesion percentage (necrosis) on the Tahiti lime flowers, the petals were initially measured lengthwise using a vernier caliper, after which a photograph was taken with a conventional digital camera in jpg format. The photos were scaled in AutoCAD^®^ V. 19 software based on the actual petal area measurements. With the image scaled, the total area of the flower was delimited, and the affected area (that with lesions) was determined and expressed as a percentage, using the following scale of severity (adapted from Lakshmi et al., 2011).

**Table:** 

Flower area affected	Level
None	0
Up to 5%	1
6–10%	2
11–20%	3
21–50%	4
More than 50%	5

With the numeric values above, apercent severity index (PSI) was calculated for the flowers, using the following formula:

PercentSeverityIndex=Sumofnumericalratings×  100Number*ofunits×Maximumgrade

^∗^flower

To arrive at a general result for the percentage of severity in the average lesions of the flowers, the following classification was used:

**Table:** 

Group	Percent severity index	Symptomatology
Highly virulent	>40	Large confluent necrotic areas
Moderately virulent	>30–40	Numerous small necrotic spots
Less virulent	<30	Few necrotic spots

**Shivakumar et al. (2016).**

### Isolation of Fungal DNA and Molecular Identification

The endophytic fungal morphospecies (EFTL-10 and EFTL-13) used in the Tahiti lime flower severity tests were seeded in new PDA media to obtain pure colonies and extract biomass from mycelia after 8 days of growth. DNA was extracted using DNeasy Powerlyzer Power Soil Kit (Qiagen) according to t22he supplier’s extraction protocol. The evaluation of the purity and quantification of the DNA extracted from the fungal samples was carried out by spectrophotometry using Nanodrop 2000c (Thermo Scientific), calculating the concentration in ng / μL, with dilutions adjusted for absorbance readings at 260 nm and the yield was calculated as the amount of DNA obtained (ng) over the weight of mycelium used for extraction (μg). The extractions were carried out in triplicate.

Markers ITS and TEF were amplified and used for taxonomic purposes. ITS region was amplified using the fungal universal primers ITS1 and ITS 4; ITS1: 5′ TCCGTAGGTGAACCTGCGG 3′—ITS4: 5′ TCCTCCGCTTATTGATATGC 3′; TEF region was amplified using the fungal universal primers TEF_983F: 5′ GCYCCYGGHCAYCGTGAYTTYAT3′ TEF_2218R:5′AT GACACCRACRGCRACRGTYTG 3′, and then capillary sequenced. ITS and TEF sequences were aligned using the MAFFT package and the alignment was imported into MEGA v7. The sequence data generated were compared against the GenBank database through BLAST^1^ to determine their most probable closely related taxa. Phylogenetic analysis using ITS datasets was conducted for the taxonomic study of the EFTL-10 and EFTL-13 isolates. A maximum-likelihood phylogenetic tree was performed using IQtree software. We used the TIM2e+I+G4 model, Ultrafast Bootstrap (Hoang et al., 2018) with 1,000 replicates.

## Results

### Isolation, Characterization, and Diversity of Endophytic Fungi

**FIGURE 1:**
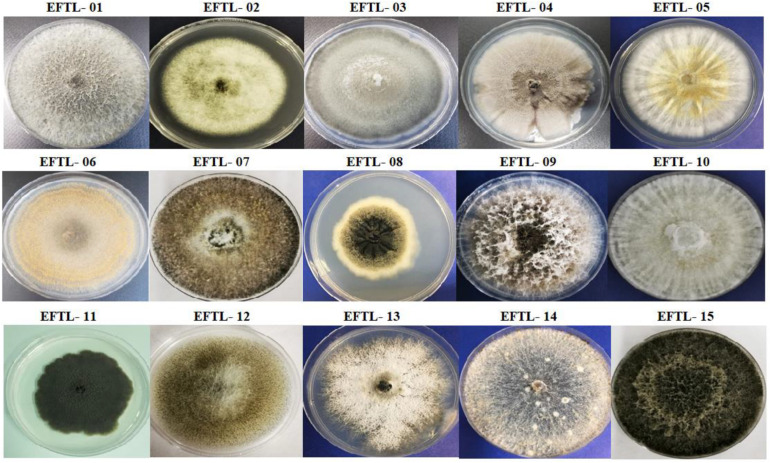
Morphospecies of endophytic fungi isolated from tissues of Tahiti lime. The identification code corresponds to the name and number of the morphospecies (Endophyte Fungi Tahiti Lime: EFTL-).

Fifteen morphospecies of endophytic fungi were isolated from the tissues of the Tahiti lime and identified using the code EFTL- (Endophytic Fungi Tahiti Lime), as illustrated in Figure 1. The endophytic fungi were identified using traditional morphological identification methods. The morphological characteristics of these fungi indicate that they belong to the Ascomycetes. Only two were classified as sterile mycelium. Fungi represented in these findings include the genera: *Phoma* sp., *Fusarium* sp., *Curvularia* sp., *Colletotrichum* sp., *Diaporthe* sp., *Verticillum* sp., *Xylaria* sp., *Nigrospora* sp., *Trichoderma* sp., *Alternaria* sp., *Phyllosticta* sp., *Chaetomium* sp., and *Micelia sterilia*.

From a total of 486 fragments of branches, leaves, and fruit seeded in PDA media after each of the 6 sampling trips carried out, 138 fungal isolates were obtained. Table 1 indicates the morphospecies identified and the number of isolates, with the relative isolation frequency for each type of vegetal tissue. It can be observed that these endophytic isolates from branches, leaves, and fruit do not always exhibit the same isolation frequency. The maximum isolation frequency was observed in leaves (41.30%), followed by branches (39.86%), and fruit (18.84%). An ANOVA test (*p* < 0.05) indicated that there are significant differences in the isolation frequencies between tissues, as is especially evident in the much lower frequency noted in fruit compared to branches and leaves.

**TABLE 1:** Number and relative frequencies of fungal isolates by colonized tissues of Tahiti lime.

Morphospecies	Number of isolations	Relative frequency percentages		
		Branches	Leaves	Fruit
EFTL-01	13	4.35	5.07	0.00
EFTL-02	5	1.45	0.72	1.45
EFTL-03*	11	3.62	2.90	1.45
EFTL-04*	12	3.62	5.07	0.00
EFTL-05	6	1.45	0.72	2.17
EFTL-06	11	4.35	2.90	0.72
EFTL-07	8	2.17	2.17	1.45
EFTL-08	14	4.35	3.62	2.17
EFTL-09	10	2.90	1.45	2.90
EFTL-10*	5	1.45	2.17	0.00
EFTL-11	10	2.17	3.62	1.45
EFTL-12*	10	1.45	4.35	1.45
EFTL-13*	9	2.17	2.90	1.45
EFTL-14	7	1.45	3.62	0.00
EFTL-15	7	2.90	0.00	2.17
Total	138	39.86	41.30	18.84

ANOVA: F = 5.064; (P = 0.01). *Morphospecies selected for antagonism tests.

The morphospecies isolated with the highest frequency from Tahiti limes were: EFTL-01; EFTL-03; EFTL-04; EFTL-06; EFTL-08; EFTL-09; EFTL-11; and EFTL-12. Nevertheless, in all of the 6 samplings undertaken, the isolates from fruit exhibited the lowest frequency (Table 1).

The diversity of endophytic fungi was observed at the level of their richness (number of taxa = *S*) and abundance (number of isolates or individuals). It was noted that although richness of fungal morphospecies was similar for both branches and leaves, it was lower in fruit. The diversity of fungi found (Table 2), using Simpson’s index, was very similar between the branches (0.92), leaves (0.91), and fruit (0.90). This is to say that a very similar distribution exists between the species sampled according to the type of tissue. The Shannon-Wiener diversity index (with reference values from 0 to 5), indicates that there is a normal level of diversity in this fungal community (branches: 2.56; leaves: 2.52; fruit: 2.35) (Table 2). With respect to this indicator, the tissues were fairly equal, except for fruit, which was somewhat lower.

**TABLE 2:** Diversity indices of endophytic fungi from tissues sampled.

	Tissue type		
Indicators	Branches	Leaves	Fruit
Individuals (isolates)	49	57	26
Speciesrichness	14	14	11
Shannon- Wiener diversity	2.56	2.52	2.35
Simpson diversity	0.92	0.91	0.90
Speciesevenness	0.92	0.89	0.95

### Antagonism Tests Against Phytopathogenic Fungi

The 15 resulting fungal morphospecies were subjected to stressful growth with low nutrient conditions in a PDA medium for 8 days (before the antagonism tests). Only the fastest-growing after 5 days (EFTL-03, EFTL-04, EFTL-10, EFTL-12, and EFTL-13) (^∗^Table 1) were selected to trial against the phytopathogen fungus *Colletotrichum acutatum* C-100.

**FIGURE 2:**
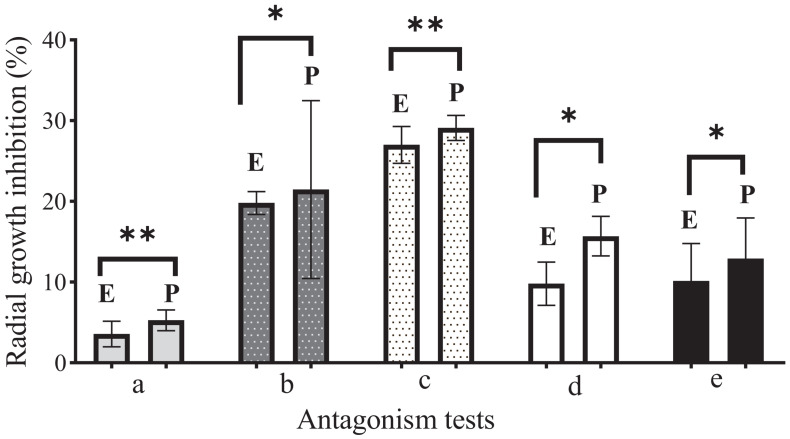
Comparison of antagonism tests during 7 days of growth. E: Endophyte. (a) EFTL-03; (b) EFTL-04; (c) EFTL-10; (d) EFTL-12; (e) EFTL-13; P: Phytopathogen (*Colletotrichum acutatun* C-10). All dual trials are significantly different (^∗^denotes *P* < 0.05) based on Bonferroni’s range test. ^∗^denotes significant differences; ^∗∗^denotes highly significant differences.

During the first 7 days of dual growth, it was observed that the average radial growth inhibition percentages of the endophytic fungi were less than those of the phytopathogenic fungi (Table 3); that is, the endophytic fungi limited the growth of the phytopathogens in the culture medium, under the same temperature and nutrient conditions. Figure 2 illustrates the comparison in the dual tests (endophyte-phytopathogen) of the 5 endophytic fungi tested (EFTL-03, EFTL-04, EFTL-10, EFTL-12, and EFTL-13). Although they grew for the same experimental time, pairs **b** and **c** had higher values for their percentage of inhibition for each fungus; nevertheless, there are significant differences between these pairs (ANOVA/Bonferroni test; *F* = 6.738; *P* = 0.0013), with the phytopathogen largely inhibited.

**TABLE 3:** Percent of radial growth inhibition of endophytic and phytopathogenic fungi (*Colletotrichum acutatum* C-100).

Dual trial	Fungal morphospecies	Type of interaction	(PIRG-E-%)	(PIRG-P-%)
A	EFTL-03	5	3.54 ± 1.6	5.26 ± 1.3
B	EFTL-04	5	19.80 ± 1.4	21.47 ± 1.1
C	EFTL-10	2++	27.00 ± 2.3	29.09 ± 1.6
D	EFTL-12	5	18.06 ± 0.2	6.38 ± 1.3
E	EFTL-13	2+	10.10 ± 0.3	12.90 ± 0.1

**Means of percentage of growth inhibition ± SD.**

**FIGURE 3:**
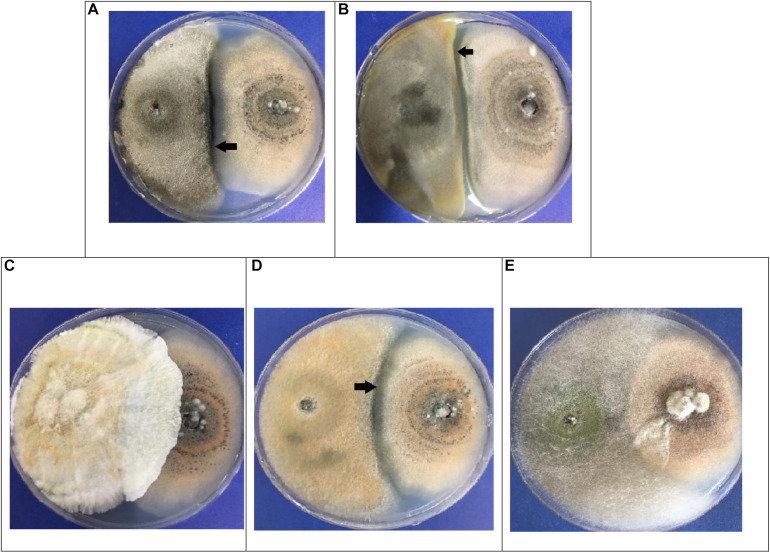
Dual *in vitro* tests for endophytic fungi (left) vs. *Colletotrichum acutatum* C-100 (right). **(A)** EFTL-03/C-100; **(B)** EFTL-04 / C-100; **(C)** EFTL-10/C-100; **(D)** EFTL-12 / C-100; **(E)** EFTL-13/C-100. The black arrows indicate the zone of limiting interaction between the two fungi (>2 mm).

When the trials were completed after 10 days, the fungi maintained a similar mycelial distribution in three of the pairs (A,B,D) as is shown in Figure 3. Here, it can be seen that both did not continue growing, but rather that they exhibited a Type-5 interaction, where the opposing fungi demonstrated similar growth but maintained a distance of > 2 mm between them (black arrows in Figure 3). The c and **e** pairs exhibited a Type-2 interaction, where the mycelium of the endophytic fungus outgrew that of the phytopathogenic. This is seen to be most true in pair **c** (2++), and slightly in pair **e** (2+) (Figure 3). Due to their performance, these two fungi were selected for the following *in vivo* tests.

### Severity Tests on Tahiti Lime Flowers

In each of the tests to evaluate the severity of *C. acutatum* C-100 on Tahiti lime flowers previously inoculated with the phytopathogen and then counter-inoculated with a 10^3^ spore/ml solution of the endophytic fungi EFTL-10 and EFTL-13, no considerable development of anthracnose-style lesions was found in the experimental units, nor in the replicas, during the observation period (72 h). In the control set only inoculated with *C. acutatum* C-100, 35.63% coverage of lesions was observed, yielding a lesion degree of 4, in each of the experimental units (Table 4).

**TABLE 4:** Percentages of floral lesions and percentage severity index (PSI).

Indicators	Treatments		
	C-100	EFTL-10 + C-100	EFTL-13 + C-100
% Lesion R1	37.36 ± 12.3	0.28 ± 0.3	1.96 ± 1.0
% Lesion R2	34.38 ± 12.1	0.30 ± 0.5	2.88 ± 2.5
% Lesion R3	35.16 ± 14.3	0.28 ± 0.4	0.72 ± 1.0
Average (%)	35.63 ± 1.5	0.29 ± 0.1	1.85 ± 1.1
Degreeofeffect	4	1	1
PSI (%)	80.00	1.90	12.40

**Means of percentage of flower lesions ± SD.**

**FIGURE 4:**
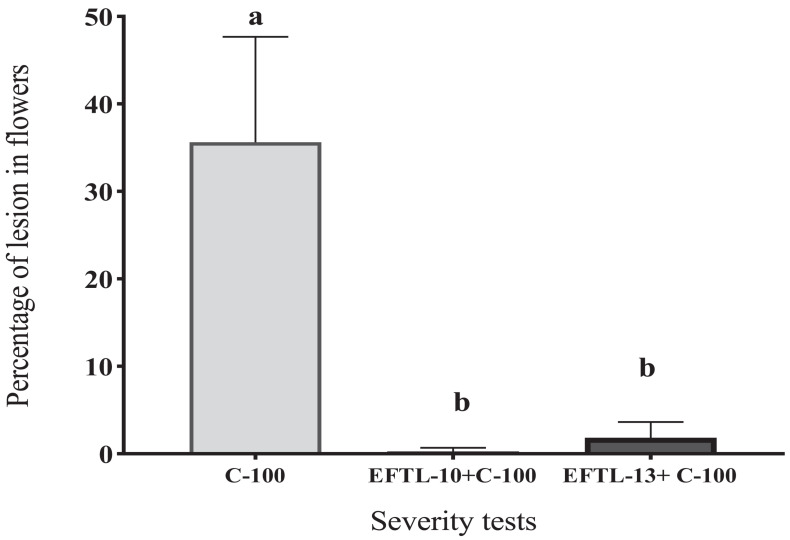
Percentage of lesions in Tahiti lime flowers. Flowers inoculated with 20 μL of spore solution to induce anthracnose: C-100 (flowers inoculated with *C.acutatum*- control lesions); EFTL-10 + C-100 (flowers inoculated with endophytic spore solution EFTL-10 + spores of *C. acutatum* at 24 h); EFTL-13 + C-100 (flowers inoculated with EFTL-13 endophyte spore solution + *C. acutatum* spores at 24 h. Bars with different letters show significant differences (*p* < 0.05).

**FIGURE 5:**
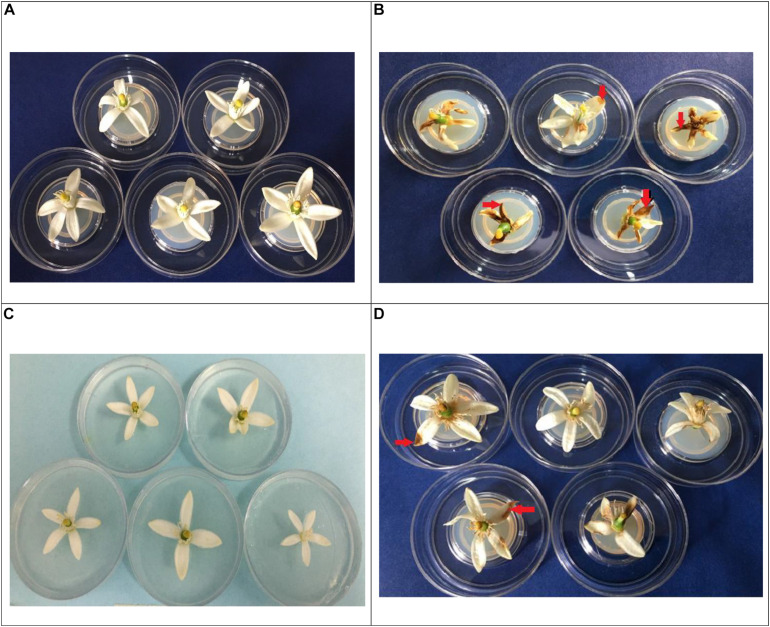
Incidence of necrosis (red arrows), in Tahiti lime flowers. *In vivo* treatments: **(A)**: flowers inoculated with distilled water control; **(B)**: flowers inoculated with 20 μL spore solution of *C. acutatum* C-100; **(C)**: EFTL-10 + C-100 (flowers inoculated with 20 μL of EFTL-10 endophyte spore solution + C-100 spore solution at 24 h; **(D)**: EFTL-13 (flowers inoculated with 20 μL EFTL-13 endophyte spore solution + C-100 spore solution at 24 h).

The flowers that were co-inoculated with the endophytic spore solutions before the application of the 10^3^ spore/ml phytopathogen solution (EFTL-10 + C-100; EFTL-13 + C-100), exhibited low percentages of lesions: 0.29 and 1.85%, respectively, yielding a lesion degree of 1 (Table 4). This data was analyzed using an ANOVA and Tukey test *aposteriori*, which demonstrated significant differences between treatments (Figure 4). Specifically, C-100 vs. EFTL-10 (*P* < 0.0001) and C-100 vs. EFTL-13 (*P* < 0.0001) and without significant difference between the two treatments (*P* = 0.8153) (Figure 4). That is, the phytopathogenic fungus was inhibited by the endophytic on the flower petals; with EFTL-10 being more effective than EFTL-13 (Figure 5).

The presence of necrotic areas on Tahiti lime flower petals is common in crops of these plants, as was observed during sample collection. In the current study, a high percentage of flowers developed necrotic stains within 72 h *in vivo* after inoculation with the phytopathogenic *C. acutatum* C-100 spores and distilled water, with a high severity index (80%) as shown in Table 4. The same index for plants inoculated with the endophytic spore solutions (EFTL-10 and EFTL-13) before inoculation with the phytopathogen was 1.9 and 12.40%, respectively. The control exerted by the endophytic fungi over the development of necrotic areas on flower petals can thus be readily observed (Figure 5).

### Molecular Identification of Antagonistic Endophytic Fungi

**FIGURE 6:**
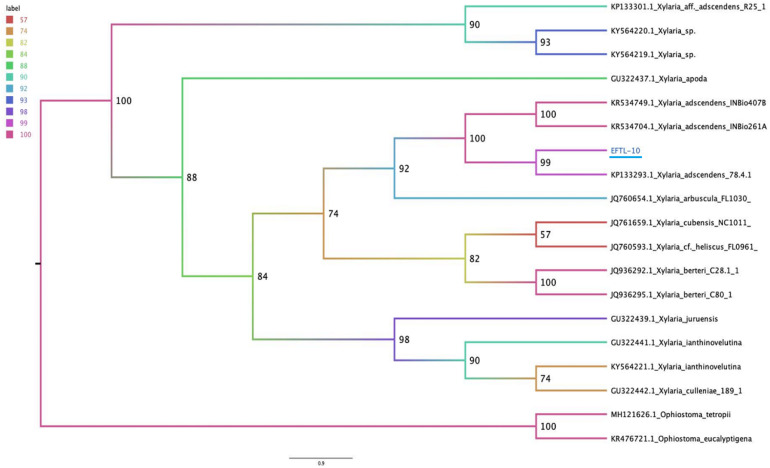
Phylogenetic tree (*Maximum likelihood*) of the ITS ribosomal intergenic region of endophytic fungus EFTL-10, performed using IQtree software. We used the TIM2e+I+G4 model, Ultrafast Bootstrap (Hoang et al., 2018) with 1,000 replicates.

**FIGURE 7:**
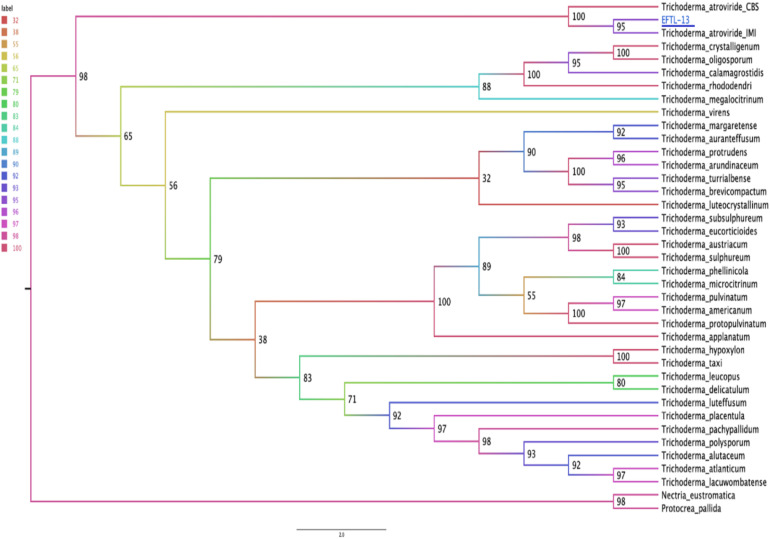
Phylogenetic tree (Maximum likelihood) for the endophytic fungus EFTL-13. Based on two concatenated markers: the ITS ribosomal intergenic region and the TEF coding gene of the microorganism. We used the TIM2e + I + G4 model, Ultrafast Bootstrap (Hoang et al., 2018) with 1,000 replicates. The consensus tree confirmed the genus and specie, *Trichoderma atroviride*.

The molecular tests applied to the morphospecies EFTL-10 and EFTL-13 resulted in consensus sequences, which were compared to the GenBank database, resulting in a finding of two possible species. The phylogenetic trees for the two species (Figures 6, 7), illustrate the relationships between the resulting sequences and those listed in GenBank. The endophytic fungus EFTL-10 was confirmed to be the species ***Xylaria adscendens***, with a 99% match to the strain KP133293.1; and the consensus tree confirmed the genus and specie, ***Trichoderma atroviride*** for EFTL-13. The distance matrix confirmed this result, showing that the lowest distance iswith *Trichoderma atroviride*, with a distance of 0.0020. Both species are taxonomically within the phylum Ascomycota, and the families Xylariaceae and Hypocreaceae, respectively.

## Discussion

### Characterization and Diversity of Endophytic Fungi From Tahiti Lime

The endophytic fungal community associated with tissues from Tahiti lime branches, leaves, and fruit in the current study was mainly composed of fungi belonging to the phylum Ascomycota, and represented by 15 morphospecies. Although studies of diversity in citrus are very rare in the literature, in the present case, the diversity of taxa found was similar to other findings. For example, in terms of endophyte species richness, our results were similar to those of Juybari et al. (2019), who found 30 fungal taxa in leaf, bark, and xylem samples of *Citrus sinensis* in Iran. Likewise, Douanla-Meli et al. (2013) recorded 20 morphospecies in 482 isolates from *Citrus limon*. In the review “Endophytic fungi reported from *Citrus* species” (Nicoletti, 2019), it can be seen that the endophytic fungi genera found in various citrus species coincide with those preliminarily reported in the present study; for example, *Alternaria* sp., *Nigrospora* sp., *Fusarium* sp., *Xylaria* sp., *Colletotrichum* sp, *Diaporthe* sp., and *Chaetomium* sp., among other endophytes.

For the frequency of isolates from plant tissues, the greatest number were found in leaf fragments (41.3%), closely followed by branches, but much fewer in fruit. This finding is also very similar to the Juybari et al. (2019) study, in which 46.2% of isolates originated from leaves, 34.8% from bark, and 19% from xylem. Although the selectivity of endophytic fungal species in colonizing different plant tissues remains under debate due to the many influencing factors in play (Mane et al., 2018), it should be noted that the presence of endophytic fungal species is ubiquitous in plant tissues, being differentiated in their frequency and diversity, and possibly based upon the symbiotic capacity that they acquire with the host plant (Selim, 2012; Fesel and Zuccaro, 2016).

The Shannon-Wiener diversity indices reported in the present study for Tahiti limes, specifically those for branches (2.56) and leaves (2.52), are similar to those found by Sadeghi et al. (2019). Those authors evaluated the spatiotemporal distribution and diversity of endophytic fungi in *Citrus reticulata*, and found the similar indices for leaves and stems, with values of 2.55 and 2.61, respectively. The Simpson indices and evenness reported by those authors were also very similar to those in the present study. However, the difference between the richness of 15 morphospecies isolated in the present study compared with the 702 endophytes isolated by Sadeghi et al. (2019), suggests that in our case, the richness may have been limited by the breadth of sampling locations chosen, as well as the agricultural species selected, and the difficulty of isolating them from vegetal tissues (Ikram et al., 2019; Fadiji and Babalola, 2020).

### *In vitro* Antagonism Tests: Endophytic Fungi vs. *Colletotrichum acutatum* C-100

In recent years, a large number of studies have reported the antagonistic effect of endophytic fungi upon phytopathogenic ones (De Silva et al., 2019; da Costa Silveira et al., 2020; Rojas et al., 2020). This effect is generally due to the production of secondary metabolites with bioactivity against these pathogens (Huang et al., 2015; Serrano et al., 2017; Latz et al., 2019). *C. acutatum* is one of the species of phytopathogenic fungi identified as a causal agent of anthracnose in citrus hosts (Damm et al., 2012; Barquero et al., 2013; Ben Hadj Daoud et al., 2019). Nevertheless, other species also exist, such as *Colletotrichum gloeosporioides*, with very similar pathogenic tendencies (Ben Hadj Daoud et al., 2019). In our study, the *in vitro* interactions of endophytic fungi against the phytopathogenic *C. acutatum* allowed us to determine that the endophytic fungi produce some substance or physiological phenomena that impedes the normal growth of the phytopathogen (Figure 2). In 3 of the 5 antagonism cases, the endophyte and the phytopathogen maintained a mutual separation in the center of the Petri dish, thus indicating that although these endophyte strains have an effect, the phytopathogen nevertheless resists them. However, in two cases, the superior growth of the endophytes indicates their greater potential as antagonists, perhaps due to the greater production of inhibitory substances.

When opposing other fungi, endophytic fungi tend to generate various effects against the antagonist (Latz et al., 2018), among these, mycoparasitism (Cao et al., 2009; Latz et al., 2018; Rajani et al., 2020), in which they directly affect the pathogen by hyphal coiling or penetration; and indirect, with only physical contact. They may also exhibit antibiosis (Wei et al., 2019), where the pathogen is inhibited by metabolites produced by the endophyte (Mousa and Raizada, 2013), and competition, where fungi compete for food, with the endophyte using the resource better (Serrano et al., 2017). Although these specific phenomena were not microscopically evaluated in the present work, the effect of endophytic fungi was evidenced *in vitro* against *Colletotrichum acutatum* as a phytopathogen (Figure 2).

The *in vitro* inhibitory effect of endophytic fungi against phytopathogenic species of anthracnose-causing *Colletotrichum* has been reported in various studies of *Colletotrichum* sp. For example, various endophytic fungi have been trialed against *C. gloeosporioides* in Amazonian plants such as guarana and açai palm, with results exhibiting inhibition by mycoparasitism and antibiosis against the pathogen (Bonatelli et al., 2016; Peters et al., 2020). Another study evaluated the effect of metabolites of the endophytic fungi *Phoma herbarum*, by isolating an alcoholic compound which demonstrated effective antagonism against *C. gloeosporioides* that cause anthracnose in *Curcuma longa* (Gupta et al., 2016). We were unable to find other works concerning the antagonism of endophytic fungi against *Colletotrichum acutatum* in citrus. However, in other species such as *Olea europaea*, endophytic fungi were isolated with inhibitory action against the growth of *C. acutatum* when cultivated together (Landum et al., 2016), thus confirming that this fungus can be controlled by mechanisms and active compounds of endophytic fungi.

### *In vivo* Tests: Inhibition of Severity in *C. acutatum* C-100

*Colletotrichum* sp. infection in citrus tissues and organs usually occurs by mechanisms that depend on the tendencies of the phytopathogen (De Silva et al., 2017; Ben Hadj Daoud et al., 2019). This can occur biotrophically, where the pathogen remains within plant tissues and absorbs plant metabolites (Vargas et al., 2012) or necrotrophically, where it actively infects and colonizes plant cells, leading to cell death and being observable as dark spots or necrosis (Peres et al., 2008; Aiello et al., 2015). The tests carried out in this work demonstrated the considerable necrotrophic effect of *C. acutatum* (C-100) on Tahiti lime flower petals at 72 h after inoculation with spore solution. The percentage of lesion coverage decreased when the petals were previously inoculated with a spore solution of the 2 selected endophytic morphospecies (EFTL-10 and EFTL-13); that is, the *C. acutatum* C-100 strain exhibited sensitivity to the presence of endophytic fungal spores, reducing its infection capacity and severity to produce necrosis in leaves.

The inhibitory properties of the two endophytic species tested (EFTL-10 and EFTL-13) may be related to one of the mechanisms described above: antibiosis, competition, or mycoparasitism. Further studies to evaluate virulence and metabolite products of fermented liquids are key in order to put forth these endophytic fungal strains as promising candidates for the control of anthracnose. This is especially so, given that the traditional manner of control for these phytopathogens involves fungicides for agriculture in general (Chen et al., 2016; Ishii et al., 2016), and in particular for citrus crops (Peres et al., 2004), a situation which is leading to resistance in *Colletotrichum* species, as reported by Forcelini and Peres (2018) for *C. acutatum* species in strawberry crops. The alterative to this conventional practice is to bring sustainable agriculture technology to citrus cultivation using biological controls, including fungal microorganisms with bioactive potential against diseases. Regarding this, it is important to consider that the application of these products derived from endophytic fungi with inhibitory capacity in phytopathogenic fungi should be applied in field trials (in the same Tahiti lime or greenhouse crops), as has been done in some studies with cucumber pathogenic fungi, where 3 isolates of endophytic fungi successfully suppressed the severity of wilt when co-inoculated with the pathogen *Fusarium oxysporum*, in greenhouse studies (Abro et al., 2019).

### Molecular Identification of the Endophytic Strains EFTL-10 and EFTL-13

The fungi EFTL-10 and EFTL-13 identified in this work, according to the GenBank search and support for phylogenetic relationships, show similarity with the species *Xylaria adscendens* and *Trichoderma atroviride*. These species of the Phylum ascomycota exhibit both free and endophyte lifestyles (Soto Medina and Bolaños Rojas, 2013; Naranjo-Ortiz and Gabaldón, 2019). Some xylaria species have been recorded as endophytes with potential for biological control of phytopathogens (Villavicencio-Vásquez et al., 2018), including in citrus plants; for example, *Xylaria cubensis* was isolated from healthy leaves, bark, and xylem of *Citrus sinensis* in different seasons and age classes in Iran (Juybari et al., 2019), and *Xylaria* sp. in *Citrus limon* (Douanla-Meli et al., 2013). Few studies were found for citrus fruits regarding the evaluation of antagonism of *Xylaria* species against *Colletotrichum* sp., except works where diversity is reported in several species (Nicoletti, 2019). However, a study similar to our current paper evaluated the inhibitory action of isolated endophytic species of *Olea europaea* L. on the growth of *Colletotrichum acutatum*, among which a morphotype of the Xylariaceae family was found, but could not be positively identified for species, that caused inhibition against the phytopathogen (Landum et al., 2016). This indicates the potential that *Xylaria adscendens* may have as a controller of *C. acutatum* in Tahiti lime.

With respect to *Trichoderma* sp., this is a very diverse genus, frequently associated with the roots of plants, which can promote biological control of phytopathogenic fungal species (Mukherjee et al., 2013; Rajani et al., 2020). Regarding biological control, a publication was found that reports results for the same species of *T. atroviride* considered in our study, in which the fungi was also evaluated as an antagonist against the phytopathogen *Fusarium solani*, finding that it is an efficient controller of the phytopathogen and significantly reducing the severity of the disease, due to the production of active compounds (Toghueo et al., 2016). Therefore, it is noteworthy that the ability to produce various metabolites and adapt to various experimental conditions, affords the fungi *Xylaria adscendens* and *Trichoderma atroviride* the possibility of being used in the biotechnology industry. As always, additional support through complementary field and laboratory research will be required to further expand the available science, and make possible their use as biological control agents against *Colletotrichum acutatum* in Tahiti lime.

## Data Availability Statement

The raw data supporting the conclusions of this article will be made available by the authors, without undue reservation.

## Author Contributions

JM-G participated in the field, laboratory, and manuscript writing activities. BG-S participated in the laboratory work and consultancy in the drafting of the document. JA participated in the review and consultancy. All authors contributed to the article and approved the submitted version.

## Conflict of Interest

The authors declare that the research was conducted in the absence of any commercial or financial relationships that could be construed as a potential conflict of interest.

## Footnotes

1 http://blast.ncbi.nlm.nih.gov/
